# A Quantitative Genetic Study of Sclerotinia Head Rot Resistance Introgressed from the Wild Perennial *Helianthus maximiliani* into Cultivated Sunflower (*Helianthus annuus* L.)

**DOI:** 10.3390/ijms23147727

**Published:** 2022-07-13

**Authors:** Zahirul I. Talukder, William Underwood, Christopher G. Misar, Gerald J. Seiler, Xiwen Cai, Xuehui Li, Lili Qi

**Affiliations:** 1 Department of Plant Sciences, North Dakota State University, Fargo, ND 58108, USA; zahirul.talukder@usda.gov (Z.I.T.); xuehui.li@ndsu.edu (X.L.); 2 USDA-Agricultural Research Service, Edward T. Schafer Agricultural Research Center, 1616 Albrecht Blvd. N., Fargo, ND 58102, USA; william.underwood@usda.gov (W.U.); christopher.misar@usda.gov (C.G.M.); gerald.seiler@usda.gov (G.J.S.); 3 USDA-Agricultural Research Service, Wheat, Sorghum and Forage Research Unit, 251 Filley Hall, 1625 Arbor Drive, Lincoln, NE 68583, USA; xiwen.cai@usda.gov

**Keywords:** sunflower, *Helianthus maximiliani*, *Sclerotinia sclerotiorum*, head rot, disease resistance, QTL mapping

## Abstract

Sclerotinia head rot (HR), caused by *Sclerotinia sclerotiorum*, is an economically important disease of sunflower with known detrimental effects on yield and quality in humid climates worldwide. The objective of this study was to gain insight into the genetic architecture of HR resistance from a sunflower line HR21 harboring HR resistance introgressed from the wild perennial *Helianthus maximiliani*. An F_2_ population derived from the cross of HA 234 (susceptible-line)/HR21 (resistant-line) was evaluated for HR resistance at two locations during 2019–2020. Highly significant genetic variations (*p* < 0.001) were observed for HR disease incidence (DI) and disease severity (DS) in both individual and combined analyses. Broad sense heritability (*H*^2^) estimates across environments for DI and DS were 0.51 and 0.62, respectively. A high-density genetic map of 1420.287 cM was constructed with 6315 SNP/InDel markers developed using genotype-by-sequencing technology. A total of 16 genomic regions on eight sunflower chromosomes, 1, 2, 10, 12, 13, 14, 16 and 17 were associated with HR resistance, each explaining between 3.97 to 16.67% of the phenotypic variance for HR resistance. Eleven of these QTL had resistance alleles from the HR21 parent. Molecular markers flanking the QTL will facilitate marker-assisted selection breeding for HR resistance in sunflower.

## 1. Introduction

Sunflower (*Helianthus annuus* L.) is one of the most important vegetable oil-producing crops worldwide. Approximately 57 million tons of sunflower seeds were produced globally in 2021 [1]. One of the most important constraints to sunflower production in the cool humid climate is the disease caused by the necrotrophic fungal pathogen, *Sclerotinia sclerotiorum* (Lib) de Bary. *S. sclerotiorum* exhibits a broad host range of over 400 dicotyledonous plant species, including many important crops, such as sunflower, oilseed rape, soybean, beans, peas, and lentils [2]. *S. sclerotiorum* attack on sunflower is manifested in several forms based on the infection site and mechanism. The wilt/basal stalk rot (BSR) initiated by root infection from growing fungal mycelia (unique to sunflower). A mid-stalk rot (MSR) is incited by germination of airborne ascospores on sunflower leaves, and a head rot (HR) caused by the germination of ascospore on sunflower capitula. The fungus can cause serious economic damage to sunflower, either directly by yield loss due to premature plant death, lodging or disintegration of heads, or indirectly by contaminating the seed lots with sclerotia and reducing their market value [3]. The prevalence of BSR and HR is more common than MSR in the United States [4]. While wilt/BSR is considered as the predominant Sclerotinia disease in the major sunflower growing areas of the United States, HR can cause serious economic losses in a season with higher-than-normal precipitation during the blooming period [5]. 

Resistance to Sclerotinia in sunflower appears to be complex, involving many small effect genes. Despite a common causal agent, negative phenotypic correlation (*r* = −16, *p* < 0.05) was observed between responses to the two forms of the Sclerotinia diseases (BSR and HR) assessed in a sunflower diversity panel [6]. It was hypothesized that there might be different sets of genes triggered in the host in response to pathogen attack incited by a different mode of infection and in different tissues of the plant [6]. Thus, breeders cannot concurrently select for resistance for one form of Sclerotinia disease and achieve resistance for another form of the Sclerotinia disease. Screening for resistance to any of the Sclerotinia diseases requires expensive, specialized field nurseries to obtain the most reliable data for selection. Special automated misting nurseries and manual ascospore inoculations are required for reliable evaluations of Sclerotinia HR resistance, while BSR field screening inoculation method involves side-dressing of *S. sclerotiorum* infected grain [7]. Inability to select for resistance to both diseases simultaneously in breeding nurseries essentially doubles the resources and effort required to combat losses due to Sclerotinia infection. 

Cultivated sunflower generally lacks resistance to Sclerotinia, although some variability in tolerance exists [6,8,9,10]. Head rot resistance quantitative trait loci (QTL) mapping efforts have been described in a few studies using biparental populations. Mestries et al. [11] first reported identification of two HR resistance QTL that together explained 38% of the phenotypic variation in the population. Gentzbittel et al. [12] reported a major HR resistance QTL on linkage group 1 (LG1) of the RFLP map, which corresponds to LG8 of the public SSR reference linkage map accepted by the sunflower community [13], explaining up to 50% of the phenotypic variability. Bert et al. [14,15] identified nine QTL associated with HR resistance on LGs 3, 5, 6, 7, 8, 9, 10, 13, and 17 of the RFLP map, which corresponds to LGs 11, 6, 13, 10, 9, 16, 2, 1, and 15 of the public SSR map, respectively, each explaining between 2.5 to 20% of the phenotypic variability. Subsequently, HR resistance QTL have also been reported in different populations on all LGs of the sunflower genome except for LGs 4, 5, and 6, each of which explained phenotypic variance ranging from 8.4 to 34.5% [16,17,18,19]. 

Association mapping analyses (AM) have also been conducted to find sunflower genomic regions harboring genes/QTL associated with HR resistance [20,21,22]. A candidate gene AM analysis detected significant association (*p* < 0.01) between the sunflower gene, *HaRIC_B* on LG11 and HR incidence which accounted about 20% reduction in HR incidence in an AM population composed of 94 inbred lines [20]. Filippi et al. [21] reported eight candidate gene markers significantly associated with HR resistance in an AM population comprising 135 inbred breeding lines. Four of these markers were previously associated with HR resistance in other studies, while the other four markers were described as novel markers that consistently showed association with HR resistance. Pogoda et al. [22] performed a genome-wide association study using a panel of 218 sunflower accessions evaluated in multiple years and locations identifying 15 genomic regions on nine sunflower chromosomes significantly associated with HR resistance.

Wild *Helianthus* species provide a diverse gene pool that may be a valuable genetic resources for the improvement of Sclerotinia resistance in cultivated sunflower [23]. *Helianthus maximiliani* Schrad., a diploid (2n = 2x = 34) perennial sunflower species, was found to be highly resistant to Sclerotinia HR [24]. Gene introgression from wild perennial sunflower species into a cultivated sunflower background is challenging and often requires crossing, backcrossing, and the use of embryo rescue techniques [25]. Over several years of pre-breeding, introgression of HR resistances from wild *H. maximiliani* has been accomplished, resulting in the release of sunflower germplasms for use in Sclerotinia HR resistance breeding [26]. In this study, we investigated the inheritance and genomic distribution of HR resistance factors in a sunflower germplasm line HR21 derived from wild perennial *H. maximiliani*. The aim of the research was to map QTL for HR resistance in a mapping population developed from crossing the germplasm line HR21 with a sunflower inbred line. The QTL mapped in this research will enhance our understanding of the molecular basis of HR resistance in sunflower. The molecular markers tightly linked to QTL are of great practical interest to sunflower breeders and can be integrated into marker-assisted selection (MAS) breeding programs for sunflower improvement against Sclerotinia HR. 

## 2. Results

### 2.1. Evaluation of HR Disease in the Field

**Figure 1:**
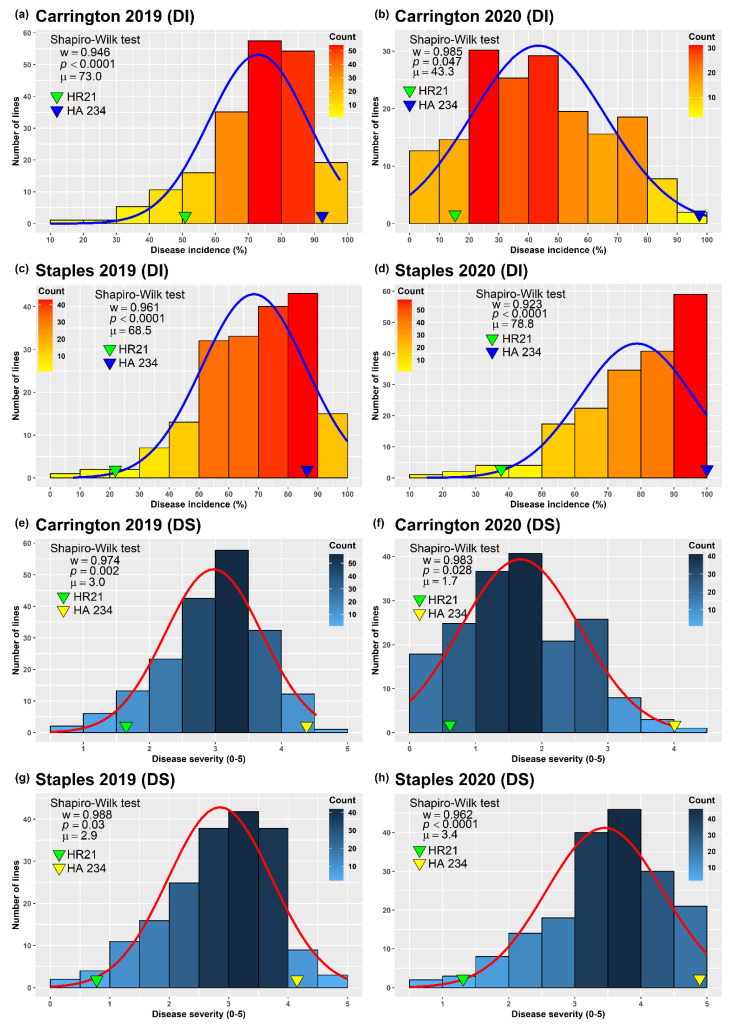
Frequency distribution of Sclerotinia head rot disease incidence, DI (**a**–**d**) and disease severity, DS (**e**–**h**) scores among sunflower F_3:4_ progeny lines derived from the cross of HA 234/HR21 evaluated at Carrington, ND and Staples, MN fields during the 2019–2020 seasons. The arrowheads indicate the values of the parent, HR21 and HA 234. The Shapiro–Wilk normality test statistic (*w*), the probability value (*p*), and the mean (μ) of the data for each environment are shown inside the respective plots.

Conspicuous HR disease symptoms consisting of tan lesions on the back of the capitulum and complete disintegration of the head for highly susceptible lines were observed in all screening trials (locations and/or years). Two parameters, disease incidence (DI) and disease severity (DS) were used to assess HR disease resistance in the population. A wide range of variation for HR DI and DS was observed in the population (Figure 1 and Figure S1). High prevalence of HR was observed in all but the Carrington 2020 environment. A clear separation of the parents, HR21 and HA 234 was observed for HR DI and DS in all environments with a mean DI of 32.3% and 94.7%, and a mean DS of 1.12 and 4.38, respectively. Among the field environments, the highest mean DI of 78.8% in the population was observed in the Staples location in 2020, ranging from 15 to 100%, followed by the Carrington location in 2019 (73.0%), ranging from 17 to 98%. The DI of Staples location in 2019 was also comparable to the Staples 2020 and Carrington 2019 trials with a mean DI of 68.5%, ranging from 8 to 100%. The lowest HR incidence was observed in Carrington in 2020 season, with a mean DI of 43.3% ranging from 0 to 100%. The DS scores of the Sclerotinia HR screening trials also followed similar trend as observed for the DI score (Figure 1 and Figure S1). The DS was the highest for the Staples location in 2020, with a mean score 3.4 ranging from 0.6 to 5.0, followed by the Carrington location in 2019, with a mean of 3.0 ranging from 0.7 to 4.5, and Staples in 2019, with a mean of 2.8 ranging from 0.3 to 5.0. Again, the lowest DS was observed at the Carrington location in 2020, with a mean value of 1.7 ranging from 0.3 to 5.0. Transgressive segregation was observed for both DI and DS in all four environments where some of the progeny lines in the mapping population showed more extreme phenotypes than either of the parents (Figure 1).

The histograms plots showed continuous distributions of the DI and DS scores ranging from highly HR resistant to highly susceptible attributes in the progeny populations (Figure 1) which supports the typical inheritance pattern of quantitative disease resistance. Shapiro–Wilk normality test revealed that both the DI and DS data in all HR screening environments were not normally distributed (Figure 1). In general, the distributions of both DI and DS data were largely skewed toward the higher values, except for the Carrington 2020 environment, which had lower disease development. 

Analysis of variances (ANOVA) of HR DI and DS data for individual environment (locations and/or years) showed highly significant (*p* < 0.001) genetic variations (data not shown). Combined analysis revealed that both genotype and environment contributed to variation for Sclerotinia HR resistance (Table 1). The genotype (G) and genotype × environment (G × E) interaction showed significant variation for both DI and DS parameters. The factors, year (Y) and location (L) and their interaction (Y × L) did not show significant effect on DI and DS. The interaction effects of genotype with year (G × Y) and genotype with location (G × L) were significant for the DS parameter, while the DI parameter only showed a significant (G × Y) interaction. 

The genetic variance estimates for both DI (93.79) and DS (0.278) were higher than the interaction components of their respective year (G × Y), location (G × L) or overall environment parameters (G × E) (Table 1). Entry means of broad sense heritability (*H*^2^) estimated across environments were 0.51 and 0.62 for DI and DS, respectively. The Spearman’s rank correlations () among HR DI and DS scores measured in the population across Carrington, ND and Staples, MN locations during 2019–2020 growing seasons are presented in Table 2. Highly significant (*p* < 0.001) positive correlations of varying extent were observed among all screening trials (locations and/or years).

### 2.2. Development of Linkage Map

Linkage analysis produced 19 LGs with 1399 unique loci and a total of 6315 SNP/InDel markers (Table 3). Chromosomes 1 and 6 produced two LGs each, while the remaining 15 sunflower chromosomes were each represented by single LG. A detailed description of the sunflower linkage map developed from the HA 234/HR21 F_2_ population is presented in Table S1. A large proportion (77.8%) of the total mapped markers co-segregated with other markers in the linkage groups. The linkage map spanned a total length of 1420.287 cM with an average density of mapped loci across the sunflower genome of 1.02 cM per locus, ranging from 0.72 to 2.59 cM per locus (Table 3). The average marker density across the sunflower genome was 0.22 cM per marker (Table 3). Individual LGs varied greatly by their length, number of mapped loci and number of mapped markers. Among them, the longest LG was LG10 (112.45 cM), followed by LGs 11 and 16, each spanning 104.36 cM. The highest number of loci were mapped on LG16 (112), followed by LG17 (108) and LG15 (107). The LGs 3, 4, 8 and 9 each had over 90 loci mapped in the linkage analysis. Chromosomes 1 and 6 splits into two separate LGs with the lowest number of mapped loci being 8, 17, 37 and 49, for LG6b, LG1b, LG6a and LG1a, respectively. Except for LG6b, all LGs had loci with co-segregating markers ranging from 56 to 89%. The highest number of markers were mapped on LG8 (616), followed by LG5 (601), LG17 (591) and LG15 (560). Like the mapped loci, the lowest number of markers were mapped on LGs 6b, 1b, 6a and 1a, with 8, 17, 84 and 49, respectively. Altogether, there were only 12 gaps over 10 cM, and 34 gaps between 5–10 cM in the entire linkage map (Table 3).

### 2.3. Identification of QTL Associated with HR Resistance

**Figure 2:**
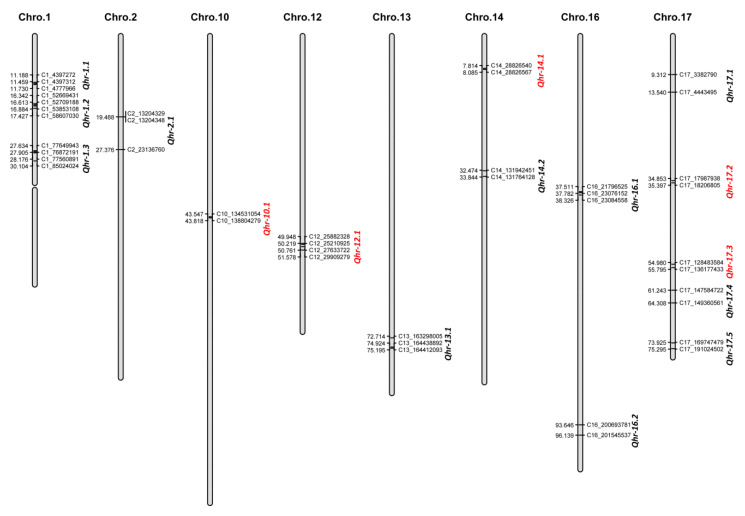
Quantitative trait loci (QTL) associated with Sclerotinia head rot (HR) resistance identified in the sunflower mapping population derived from the cross of HA 234/HR21 evaluated at Carrington, ND and Staples, MN fields during 2019–2020. The QTL where HR resistance alleles derived from the susceptible parent HA 234 were indicated in red fonts.

Significant QTL for Sclerotinia HR resistance were detected in all screening trials, as well as in the integrated environments (locations and/or years). HR resistance QTL were detected in a total of 16 genomic regions on eight sunflower chromosomes with LOD values ranging from 3.01 to 13.64 (Table 4 and Figure 2). The highest number of five QTL was detected on chromosome 17, followed by three QTL on chromosome 1 and two QTL each on chromosomes 14 and 16. The percent of the total phenotypic variance (*R*^2^) explained by each of these QTL were small to moderate, ranging from a minimum of 3.97% to a maximum of 16.67% in the mapping population. Both the parents contributed to the HR resistance in this population with eleven QTL, *Qhr-1.1*, *Qhr-1.2*, *Qhr-1.3*, *Qhr-2.1*, *Qhr-13.1*, *Qhr-14.2*, *Qhr-16.1*, *Qhr-16.2*, *Qhr-17.1*, *Qhr-17.4*, and *Qhr-17.5*, exhibiting HR resistance alleles derived from the resistant HR21 parent, while the remaining five QTL, *Qhr-10.1*, *Qhr-12.1*, *Qhr-14.1*, *Qhr-17.2*, and *Qhr-17.3*, had resistance alleles derived from the susceptible HA 234 parent. Except for *Qhr-14.1*, all the QTL reported represented both the DI and DS parameters of the HR resistance and were detected in either both years and locations or in the combined analysis. The QTL *Qhr-14.1* on chromosome 14 was detected for DS only in the Carrington 2019 environment. A detailed description of these QTL detected in different trials and across environments (locations and/or years) is presented in Table S2.

## 3. Discussion

Understanding the genetic basis of quantitatively inherited disease resistance has been one of the major challenges in crop variety development [27], particularly for Sclerotinia resistance in sunflower. Use of host genetic resistance is the most economical and environmentally friendly approach for Sclerotinia disease management in sunflower and chemical control of HR is impractical due to the difficulty of applying fungicides to the face of the sunflower head where infection begins. Consequently, identification of novel resistance loci introgressed from wild sunflower relatives will support breeding for improvement of genetic resistance to HR. The resistance donor parent in the current study is a pre-breeding germplasm with Sclerotinia HR resistances introgressed from a wild perennial *H. maximiliani* species [26]. This research effort was designed to molecularly dissect the resulting HR resistance with respect to genomic distribution, magnitude, and nature of expression under varying environments.

Despite our effort to maintain a high disease pressure throughout all field screening trials, the mean DI and DS for the Carrington 2020 environment were comparatively lower than the other three environments (Figure 1). We considered that the lower HR disease development at the Carrington 2020 environment could be attributed to some factors critical for optimum disease development that was beyond our control. Nonetheless, even in the least favorable environment for disease development, a broad range of disease incidence was observed, indicating sufficient disease pressure. The combined ANOVA of the Sclerotinia HR DI and DS scores showed significant genotype-by-environment (G × E) interactions (Table 1). However, the variance estimates of the genetic component of DI and DS in the mapping population were higher than the variance estimates of respective G × E interaction components, suggesting that the observed HR phenotype across the screening environments were mostly contributed by the genetic makeup of the progeny lines. The moderate broad sense heritability (*H*^2^) estimates of DI and DS across environments corroborate the notion that improvement of HR resistance is achievable through genetic manipulation. Despite significant G × E interactions, the Spearman’s rank correlations among the DI and DS scores measured in different environments were highly significant with varying magnitudes (Table 2), suggesting a strong consistency among the field evaluation trials of the mapping population across environments.

The phenotypic distribution of DI and DS were continuous (noncategorical) in the population in all environments, ranging from highly resistant to highly susceptible response, a typical manifestation of quantitative disease resistance (Figure 1). Transgressive segregation was observed for both DI and DS in all four environments, where some of the progeny lines of the mapping population showed more extreme phenotypes than either of the parents, suggesting that both the parents of the mapping population contributed to the HR resistance.

We used the high-throughput and cost-effective GBS technology for genotyping of the mapping population for linkage map construction and subsequently QTL analysis of the HR resistance. Even after following several stringent filtering options (see methods), a large number of high-quality polymorphic markers were available for linkage analysis. The current linkage map with 6315 SNP/InDel markers mapped on 17 sunflower chromosomes is by far the densest linkage map ever developed in sunflower using a single biparental mapping population. The length of the linkage map is 1420.287 cM, comparable to the sunflower consensus linkage map developed using three F_2_ populations [28], and also to some of the recent linkage maps developed using SNP markers [29,30,31,32,33,34,35]. Overall, our linkage map covered over 96% of the HanXRQr2.0 sunflower reference genome [36] with a minimum coverage of 89% in chromosome 14 to the maximum coverage of almost 100% in chromosomes 1, 4, 13 and 16 (Table S3). The quality of our linkage map was validated by analyzing Spearman’s rank correlations (ρ) between marker order in the linkage map with the physical positions of the respective markers on the HanXRQr2.0 reference genome. Except for LGs 1, 5 and 6 with ρ values 0.76, 0.88 and 0.81, respectively, the remaining 14 LGs showed almost perfect collinearity (ρ ≈ 1.0) of the mapped marker orders with the reference genome, indicating the high quality and accuracy of the linkage map (Table S3). Almost every marker of the linkage map corroborates with the assignment to their respective chromosome number with only 0.52% (33 of 6315) markers showing incongruity with the physical map or mapped to different LGs (Table S3). 

Despite the large number of mapped markers, the number of unique loci in the linkage map was comparatively low (1399) relative to recently published linkage maps developed using wild annual sunflower species [34,35]. A vast majority of the mapped markers (77.8%) co-segregated with other markers in the current linkage map developed from the HA 234/HR21 population. One possibility is that the number of recombination events captured in an F_2_ population comprising only 188 progeny lines is not adequate to resolve the grouping of such a large number of markers. Another possibility is that the HR21 parent, an introgression line developed from wild perennial *H. maximiliani*, had limited meiotic recombination with the regular sunflower genome in the other parent. Suppression of recombination may have occurred between chromosomes of cultivated and wild sunflower in the F_2_ population during meiosis, resulting in clustering of large number of co-segregating loci [37,38]. Additionally, short sequence reads derived from introgressed regions may not have been mapped to the XRQ reference genome due to dissimilarity to the reference *H. annuus* sequences.

Elucidating the genetics of Sclerotinia HR resistance introgressed from wild perennial *H. maximiliani* is of immense value for sustainable sunflower production, as well as diversifying the narrow genetic base of cultivated sunflower [39]. Due to the significant G × E interactions observed for the traits, we performed QTL analysis of DI and DS data collected in individual environments, as well as combined analysis across years and locations using extracted best linear unbiased predictor (BLUP) values. Overall, 16 QTL associated with HR resistance were detected in the current study on eight sunflower chromosomes, 1, 2, 10, 12, 13, 14, 16, and 17 (Table 4, Figure 2). Eleven of these QTL had alleles conferring increased HR resistance derived from the resistant HR21 parent, while the remaining five QTL had HR resistance alleles derived from the susceptible HA 234 parent. This explains the transgressive phenotype observed in some progeny lines relative to either of the parent of the mapping population which resulted from recombination of resistance/susceptible complementary alleles and generated extreme phenotypes [40]. Except for *Qhr-14.1* on chromosome 14, all QTL were simultaneously detected for both the DI and DS parameters used to assess the HR resistance in the population (Table 4). The Spearman’s rank correlations revealed that these two parameters measured within each environment were strongly correlated with ρ values of 0.95, 0.99, 0.96, and 0.98 for Carrington 2019, 2020 and Staples 2019, 2020, respectively.

Sclerotinia HR is by far the most widely studied necrotrophic sunflower disease and many QTLs have been reported in the past. Earlier HR resistance QTL studies in sunflower also detected QTL on the sunflower chromosomes where we detected QTL in the current study [12,14,15,16,17,19]. Most of these studies were published before the release of the two sunflower reference genomes currently available for use by the sunflower community (https://www.heliagene.org/HanXRQ-SUNRISE, accessed on 5 July 2022). Knowledge of common markers between linkage maps and current SNP maps allows one to compare positions between significant markers in our study and in previous studies. Understandably, no precise comparison of our map to the QTL positions of those studies can be accomplished because of the lack of common loci or sequence information of the mapped markers. However, this is not the case with some of the more recent association mapping (AM) studies of Sclerotinia HR resistances conducted after the release of the sunflower reference genomes [36,41]. Many significant markers associated with Sclerotinia HR resistance have been reported in these AM studies [20,21,22], some of these loci were mapped to chromosomes where we identified HR resistance QTL. Interestingly, none of these loci positions overlap the genomic locations with the QTL positions in the current study. This is not surprising as the HR resistance in the HR21 parent has been introgressed from a novel source of the wild perennial *H. maximiliani* species. Biparental linkage mapping has the power of detecting rare QTL alleles enabling large phenotypic effect segregating in crosses between two highly divergent parents, as in the case of our study. Conversely, AM has the advantage of surveying large number of germplasms at a higher mapping resolution and finds the most common alleles responsible for the phenotypic diversity found in natural populations; however, it has low or no power to detect rare QTL alleles [42]. In this way, a certain level of overlap is anticipated with linkage studies, but complete concordance is not. 

The sunflower sequences flanking the HR resistance QTL presented in this study will promote development of molecular markers that can aid future marker-assisted selection programs, thus complementing field-based phenotyping. The current study has provided the sunflower community with new tools to combat the devastating losses caused by the Sclerotinia HR disease. 

## 4. Materials and Methods

### 4.1. Plant Materials

A QTL mapping population consisting of 188 F_3:4_ progeny lines was developed from a cross between HA 234 and HR21. HA 234 (PI 599778) is an oilseed sunflower maintainer line susceptible to HR. HR21 is a HR resistance germplasm line selected from ‘HR MAX 1018+1323’ (PI 688642) released in 2017 by the USDA-ARS and North Dakota Agricultural Experiment Station at Fargo, ND (https://www.ag.ndsu.edu/fss/ndsu-varieties/fact-sheets-and-brochures/ReleaseofTwoSclerotiniaHeadRot.pdf, accessed on 5 July 2022). HR resistance in HR21 was derived from the wild perennial sunflower species *H. maximiliani* MAX 1018 and MAX 1323 accessions obtained from Agriculture and Agri-Food Canada, Morden, Manitoba [43]. 

### 4.2. Inoculum Preparation and Inoculation

To produce *S. sclerotiorum* ascospores for inoculations, sclerotia of isolate NEB-274 were surface sterilized in a 20% bleach solution for 4 min, rinsed with water, incubated 1 min in 70% EtOH and rinsed again with water. Sterilized sclerotia were placed in sterile, moist sand in glass Petri dishes, with 20 sclerotia per dish, and sealed with laboratory film. Petri dishes were incubated in a tissue culture chamber fitted with Reptisun UV lamps for six weeks with constant illumination at 16 °C and sterile water was added to the dishes weekly to maintain moisture. Upon uncovering Petri dishes, ascospores were collected on aluminum foil discs, desiccated 16 h in a bell jar with anhydrous calcium sulfate desiccant, and stored at −80 °C prior to use. Ascospores were suspended in spring water at a concentration of 5 × 10^3^ ascospores mL^–1^ as determined by a hemocytometer count. Each sunflower head was inoculated with 5 mL of ascospore suspension using a hand sprayer at R5.6 to R5.9 growth stages when the plants are highly susceptible to HR [44]. To provide a conducive environment for HR disease development, automatic mist irrigation was applied. Duration and frequency of misting was based on forecasted weather conditions at the time of each inoculation period. High temperature and low humidity conditions required the system to mist plants every 15 min to keep the front of the head moist. Misting continued for an additional 1 to 2 weeks after the last inoculation.

### 4.3. Experimental Design and Field Evaluation

The F_3_ and F_4_ progeny lines (n = 188) of the mapping population along with the parents and checks were evaluated for HR resistance in the summer of 2019 and 2020 at the Carrington, ND (47.507431°, −99.117550) and Staples, MN (46.385003°, −94.799661°) HR screening nurseries equipped with mist-irrigation systems. Commercial oilseed hybrid Croplan 305 and inbred line HA 441 were used as the resistant checks in all field trials. Cargill 270 and HA 89 were the susceptible checks. The experiments were laid out in a randomized complete block design with 3 replications, except for the 2019 Staples trial, which had only 2 replications. Each experimental unit consisted of a 6 m-long (Staples location) or 4.5 m-long (Carrington location) single row plot with 75 cm spacing between rows. Plants were thinned to a final stand of 15 to 20 plants per row. At the physiological maturity, individual plots were assessed for HR disease symptom on the inoculated heads using a 0 to 5 scale for DS (0 = no symptom; 1 = 1/4 of the head visibly rotted; 2 = 1/2 of head affected; 3 = 3/4 of head affected; 4 = entire head rotted, but intact; 5 = head disintegrated and no seed left), which was calculated as the mean of disease scores within each plot. Disease incidence (DI) was measured as the percentage of plants showing HR disease symptoms within each plot.

### 4.4. Data Collection and Statistical Analysis

Data were recorded for HR DI and DS for the parents, checks, and progeny lines. The phenotypic data collected in the field were analyzed for normality using Shapiro–Wilk normality test [45] and phenotypic frequency distributions were plotted using statistical package R v3.4.3 [46]. Spearman’s rank correlation among HR disease data collected in different field environments was also carried out using the R software [46].

Analysis of variance (ANOVA) of the HR DI and DS data of individual environments were performed using a generalized linear mixed model (Proc GLIMMIX) of SAS v9.4 [47]. The combined ANOVA was carried out using a mixed effects linear model (Proc MIXED) of SAS with genotype considered fixed, and all other effects considered random. The model is described as:

$$Y_{ijkl}=\mu +g_{i}+r{\left(yl\right)}_{jkl}+y_{k}+l_{l}+yl_{kl}+gy_{ik}+gl_{il}+gyl_{ikl}+e_{ijkl}$$

where $Y_{ijkl}$ is the observed response of the *i^th^* genotype in the *j^th^* replication nested in the *l^th^* location of the *k^th^* year, μ is the overall mean response, $g_{i}$ is the genetic effect of the *i^th^* genotype, $r{\left(yl\right)}_{jkl}$ is the effect of the *j^th^* replication in the *k^th^* year and *l^th^* location, $y_{k}$ is the effect of the *k^th^* year, $l_{l}$ is the effect of the *l^th^* location, $yl_{kl}$, $gy_{ik}$, and $gl_{il}$ are the first-order interaction effects, $gyl_{ikl}$ is the second-order interaction effect, and $e_{ijkl}$ is the random experimental error. Broad-sense heritability of an entry mean basis was estimated as follows [48]:

$$H^{2}=\sigma_{g}^{2}/(\sigma_{g}^{2}+\sigma_{gk}^{2}/k+\sigma_{gl}^{2}/l+\sigma_{gkl}^{2}/kl+\sigma_{e}^{2}/klr)$$

where $\sigma_{g}^{2}$ is the genetic variance, $\sigma_{gk}^{2}$ is the genotype × year variance, $\sigma_{gl}^{2}$ is the genotype × location variance, $\sigma_{gkl}^{2}$ is the genotype × year × location variance, $\sigma_{e}^{2}$ is the error variance, *k* is the number of years, *l* is the number of locations and *r* is the number of replications. 

### 4.5. DNA Extraction and Single Nucleotide Polymorphism (SNP) Genotyping

The parents and 188 F_2_ individuals of the HA 234/HR21 sunflower population were grown in the greenhouse and leaf tissue samples were collected from 4-week-old seedlings and freeze-dried. Genomic DNA was extracted from ~50 mg of freeze-dried leaf tissue using a Qiagen DNeasy 96 plant kit (Qiagen, Valencia, CA, USA) with a minor modification of the manufacturer’s protocol following Horne et al. [49]. A NanoDrop 2000 Spectrophotometer (Thermo Fisher Scientific, Waltham, MA, USA,) was used to check the quality and the quantity of the extracted DNA. Genotyping of the population was performed using genotyping-by-sequencing (GBS) technology at the University of Minnesota Genomics Center, Minneapolis, MN, USA. 

Dual-indexed GBS libraries were prepared for each sample using *Eco*T22I enzyme digestion. All libraries were combined into a single pool and sequenced across 1.5 lanes of an Illumina NextSeq HO 1 × 150-bp run which generated about ≈400 M reads for the pool. All expected barcodes were detected except those for sample 18-005-417, which was removed from further analysis. Fastq files were evenly subsampled down to a maximum of 5,000,000 reads per sample. Quality of data in fastq files was assessed with FastQC (https://www.bioinformatics.babraham.ac.uk/projects/fastqc/, accessed on 5 July 2022). Trimmomatic [50], a flexible read trimming tool for Illumina NGS data, was used to trim 3′ adapter sequences and low-quality bases from the ends of reads. Burrows–Wheeler Aligner program [51] was used to align reads to sunflower HanXRQr2.0 reference genome [36] (https://www.heliagene.org/HanXRQ-SUNRISE, accessed on 5 July 2022). The bam alignment files were sorted and indexed with Samtools [52]. Regions of bam files with more than 500 reads were down sampled to a depth of 500 reads using VariantBam [53]. Freebayes [54] was used to call variants jointly across all samples using the options ‘–genotype-qualities –min-coverage 376’. The raw VCF file generated by Freebayes was filtered to remove the lowest quality variants using vcffilter with the options ‘-f’ “QUAL > 20”. Samples with >50% missing genotypes, variants with genotype calls in less than 95% of samples, and variants with MAF < 1% were removed resulting in a raw VCF variant file containing 187 samples and 41,018 SNP/InDel markers. The markers were given a prefix of ‘C’ with their respective chromosome numbers (1 to 17) corresponding to the 17 sunflower chromosomes/LGs followed by a number being the physical position of the marker on the HanXRQr2.0 genome assembly. 

### 4.6. Linkage Mapping

The SNP/InDel markers generated by the analysis pipeline were further filtered by several criteria for linkage analysis: (a) monomorphic markers, (b) >10% missing genotype, (c) missing genotype for parents, (d) heterozygous parental genotype, and (e) markers distorted (*p* < 0.05) from expected 1:2:1 segregation ratio, were removed. The genetic linkage map was constructed using the Linux based software, Lep-MAP3, which uses maximum-likelihood algorithm capable of handle a large number of markers [55]. First, the parental genotypes were called using the ParentCall2 module of the software. The Filtering2 module was then used to remove non-informative markers and distorted markers with significant segregation distortion (dataTolerance = 0.05) leaving a total of 6340 markers for the final linkage analysis. The SeparateChromosomes2 module was used to categorize markers into linkage groups (LGs) by computing all pairwise logarithm of odds (LOD) scores between markers. An optimized LOD threshold of 29 (lodLimit = 29) seemed to provide the best result for marker clustering with 19 major linkage groups that showed good correspondence with sunflower chromosome information of the genome. The JoinSingles2All module was used to assign ‘singular markers’ into existing LGs generating a new map file with additional markers. Finally, the OrderMarkers2 module was used with the default parameter to order the mapped markers within each LG by maximizing the best likelihood positions of the markers. A Kosambi mapping function [56] was used for conversion of recombination frequencies into map distances (cM). Linkage maps were drawn using MapChart v2.2 [57].

### 4.7. QTL Mapping

QTL analyses of HR DI and DS were carried out using data from both individual environments, as well as combined analysis for locations and two years of data. Two software were used for QTL analysis in this study. First, the composite interval mapping (CIM) module of WinQTL Cartographer v2.5 was used for initial QTL analyses [58,59]. In the CIM analysis, a window size of 10 cM with a walk speed of 1 cM was chosen to scan the sunflower genome for HR resistance QTL. The forward and backward regression method (model 6) was selected with parameter set for automatic selection of five control markers for the analysis. Significance LOD threshold for independent QTL run was determined using 1000 times permutation tests [60].

A second QTL analysis software, QTL IciMapping v4.1 [61] was used to compare the results obtained from WinQTL Cartographer. We chose the inclusive composite interval mapping (ICIM) module of the software to perform the QTL analysis. The two-step mapping strategy implemented in this module proved to be more efficient for background control in detecting significant QTL associated with the trait under study [62,63]. The module begins with a stepwise regression analysis to identify the most-significant regression variables, followed by a composite interval mapping using phenotypes adjusted by the markers identified in the stepwise regression analysis step [62,63]. QTL detected in both software with significant LOD values were reported. For each significant QTL, a 95% confidence interval was calculated to obtain the QTL flanking region using a 1-LOD support interval of the most likely QTL peak position. HR resistance QTL identified in the current study were named following the convention proposed by Talukder et al. [31]. The naming of each QTL started with a prefix Q for QTL, a two-letter descriptor of the trait under study (HR), the LG number, followed by a sequential number of the QTL identified in that LG for the trait. A 400 bp sequence flanking each of the SNP/InDel markers significantly associated with the HR resistance QTL in the current study are presented in Supplementary Table S4.

## Figures and Tables

**Table 1:** Combined analysis of variance of Sclerotinia head rot disease incidence (DI) and disease severity (DS) scores among sunflower F_3:4_ progeny lines derived from the cross of HA 234/HR21 tested at Carrington, ND and Staples, MN fields during the 2019 and 2020 seasons.

Covariance Parameter	df	DI (%)			DS (0–5 Scale)		
		Estimate	F/Z Value	Pr > F/Z	Estimate	F/Z Value	Pr > F/Z
Genotype (G)	193	93.79	**2.20**	**<0.0001**	0.278	**2.79**	**<0.0001**
Year (Y)	1	0.0	-	-	0.0	-	-
Location (L)	1	0.0	-	-	0.065	0.12	0.4507
Environment (E), Y × L	1	240.66	1.22	0.1116	0.509	0.99	0.1612
Rep (Y × L)	7	2.09	1.10	0.1357	0.011	1.48	0.0693
G × Y	187	50.18	2.87	0.0020	0.081	2.64	0.0042
G × L	193	19.46	1.31	0.0956	0.063	2.15	0.0156
G × E	185	75.98	3.99	<0.0001	0.128	3.69	0.0001
Residual	1304	275.08			0.529		

F, Fisher’s F-test statistic (shown in bold); Z, Z-test statistic variances.

**Table 2:** Spearman’s rank correlations () of Sclerotinia head rot disease incidence (DI) and disease severity (DS) scores among sunflower F_3:4_ progeny lines derived from the cross of HA 234/HR21 tested at Carrington, ND and Staples, MN fields during the 2019 and 2020 seasons.

		DI (%)				DS (0–5)		
		Carrington 2019	Staples 2019	Carrington 2020	Staples 2020	Carrington 2019	Staples 2019	Carrington 2020
DI	Staples 2019	0.36 ***						
	Carrington 2020	0.24 **	0.29 ***					
	Staples 2020	0.22 **	0.47 ***	0.51 ***				
DS	Carrington 2019	0.95 ***	0.44 ***	0.30 ***	0.37 ***			
	Staples 2019	0.32 ***	0.96 ***	0.35 ***	0.54 ***	0.42 ***		
	Carrington 2020	0.25 ***	0.30 ***	0.99 ***	0.53 ***	0.31 ***	0.36 ***	
	Staples 2020	0.19 **	0.47 ***	0.52 ***	0.98 ***	0.34 ***	0.55 ***	0.54 ***

** Significant at the 0.01 probability level, *** Significant at the 0.001 probability level.

**Table 3:** Summary of sunflower linkage map developed using SNP/InDel markers in an F_2_ population derived from the cross of HA 234/HR21.

Linkage Group	Map Length (cM)	No. of Loci	No. of Markers	cM/Locus	cM/Marker	No. of Large Gaps	
						5–10 cM	>10 cM
LG1a	35.467	49	183	0.72	0.19	1	0
LG1b	22.867	17	39	1.35	0.59	2	0
LG2	82.321	60	280	1.37	0.29	3	2
LG3	83.074	91	444	0.91	0.19	5	0
LG4	97.068	91	325	1.07	0.30	2	0
LG5	71.463	65	601	1.10	0.12	2	1
LG6a	36.703	37	84	0.99	0.44	0	0
LG6b	20.726	8	8	2.59	2.59	0	0
LG7	85.532	76	376	1.13	0.23	1	2
LG8	72.924	97	616	0.75	0.12	2	0
LG9	95.238	95	324	1.00	0.29	4	1
LG10	112.454	77	396	1.46	0.28	1	2
LG11	104.361	82	268	1.27	0.39	1	2
LG12	71.316	75	284	0.95	0.25	1	0
LG13	86.061	81	300	1.06	0.29	3	1
LG14	83.451	71	252	1.18	0.33	4	1
LG15	77.431	107	560	0.72	0.14	1	0
LG16	104.355	112	384	0.93	0.27	1	0
LG17	77.475	108	591	0.72	0.13	0	0
Total	1420.287	1399	6315	1.02	0.22	34	12

**Table 4:** Summary of quantitative trait loci identified for Sclerotinia head rot disease incidence (DI) and disease severity (DS) in the sunflower population derived from the cross of HA 234/HR21.

QTL	Trait	Chro.	Position (cM)	LOD Range	Flanking Markers		*R*^2^ Range	Resistance Source
					Left	Right		
*Qhr-1.1*	DI, DS	1	11.2–11.7	3.79–7.77	C1_4397272	C1_4777966	5.22–11.09	HR21
*Qhr-1.2*	DI, DS	1	16.4–16.9	4.11–10.21	C1_52669431	C1_58607030	10.30–15.10	HR21
*Qhr-1.3*	DI, DS	1	27.9–29.2	3.40–7.23	C1_77649943	C1_85024024	4.13–10.64	HR21
*Qhr-2.1*	DI, DS	2	20.5	3.73–6.12	C2_13204329	C2_23136760	5.34–9.88	HR21
*Qhr-10.1*	DI, DS	10	43.6	3.87–6.81	C10_134531054	C10_138804279	5.01–8.78	HA 234
*Qhr-12.1*	DI, DS	12	50.2–51.3	4.37–7.45	C12_25882328	C12_29909279	8.85–13.09	HA 234
*Qhr-13.1*	DI, DS	13	73.7–75.0	3.67–5.18	C13_163298005	C13_164412093	5.63–8.82	HR21
*Qhr-14.1*	DS	14	8.0	4.29	C14_28826540	C14_28826567	5.85	HA 234
*Qhr-14.2*	DI, DS	14	32.5	3.16–11.01	C14_131942451	C14_131764128	5.49–16.35	HR21
*Qhr-16.1*	DI, DS	16	37.6–38.1	3.01–4.35	C16_21796525	C16_23084558	4.73–7.17	HR21
*Qhr-16.2*	DI, DS	16	94.7–96.1	3.34–4.25	C16_200693781	C16_201545537	4.30–5.52	HR21
*Qhr-17.1*	DI, DS	17	9.6	4.48–5.14	C17_3382790	C17_4443495	6.30–5.09	HR21
*Qhr-17.2*	DI, DS	17	35.2	3.18–13.64	C17_17987938	C17_18206805	5.13–14.60	HA 234
*Qhr-17.3*	DI, DS	17	55.3	3.49–12.31	C17_128483584	C17_136177433	5.23–11.26	HA 234
*Qhr-17.4*	DI, DS	17	64.1	3.28–5.50	C17_147584722	C17_149360561	3.97–11.26	HR21
*Qhr-17.5*	DI, DS	17	74.9	3.36–6.88	C17_169747479	C17_191024502	9.11–16.67	HR21

LOD—log of odds; *R*^2^—phenotypic variation explained.

## Supplementary Materials

The following are available online at https://www.mdpi.com/article/10.3390/ijms23147727/s1.
