# Supplementary figures and images for: A Quantitative Genetic Study of Sclerotinia Head Rot Resistance Introgressed from the Wild Perennial Helianthus maximiliani into Cultivated Sunflower (Helianthus annuus L.)
Source: Int J Mol Sci. 2022 Jul 13;23(14):7727. doi: 10.3390/ijms23147727 (PMC9321925; doi:10.3390/ijms23147727)

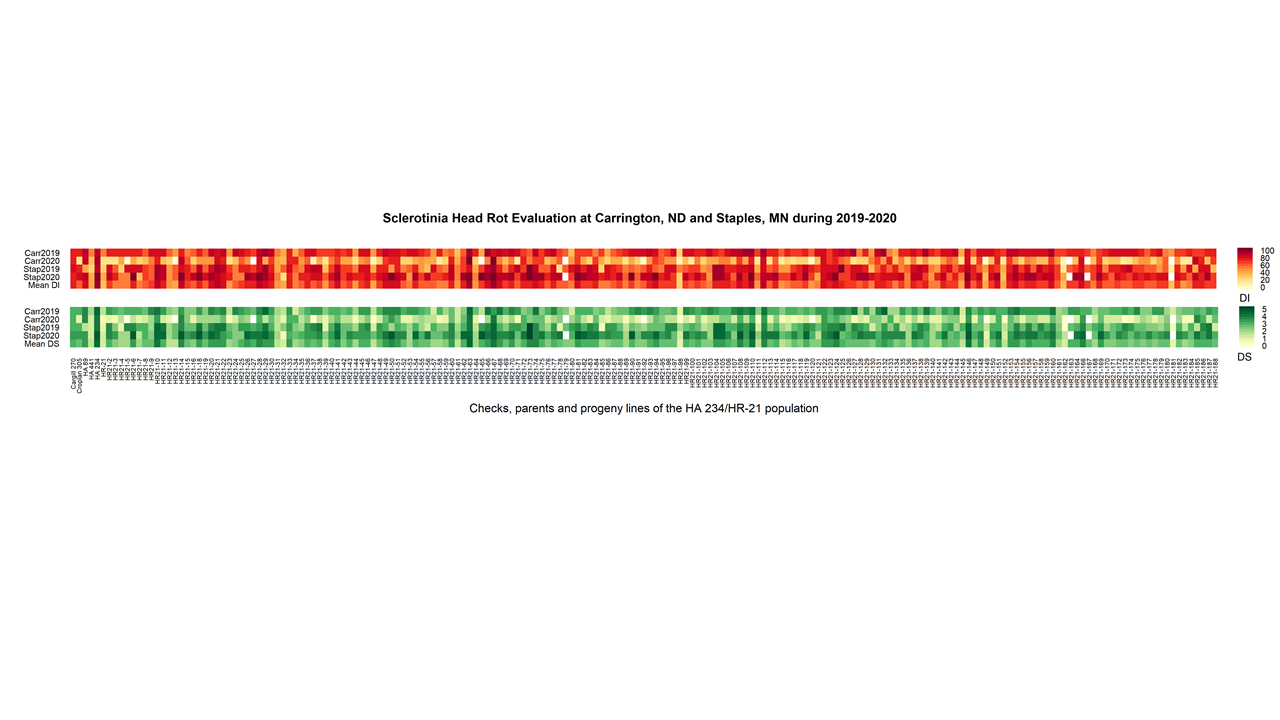

Supplement: Supplementary file 1 [file ijms-23-07727-s001.zip › Figure S1.tif]
